# A Colorimetric pH Sensor Based on *Clitoria* sp and *Brassica* sp for Monitoring of Food Spoilage Using Chromametry

**DOI:** 10.3390/s19214813

**Published:** 2019-11-05

**Authors:** Noor Azizah Ahmad, Lee Yook Heng, Faridah Salam, Mohd Hazani Mat Zaid, Sharina Abu Hanifah

**Affiliations:** 1 Center for Advanced Materials and Renewable Resources, Faculty of Science and Technology, Universiti Kebangsaan Malaysia, Bangi 43600, Selangor, Malaysia; noorazizah.ahmad@gmail.com (N.A.A.); leeyookheng@yahoo.co.uk (L.Y.H.); zani@ukm.edu.my (M.H.M.Z.); 2 Malaysian Agricultural Research Development Institute, Serdang 43400, Selangor, Malaysia; fafaridahs@mardi.gov.my

**Keywords:** colorimetric pH sensor film, mixed natural dye, ι-carrageenan, food spoilage

## Abstract

A developed colorimetric pH sensor film based on edible materials for real-time monitoring of food freshness is described. The mixed natural dyes from edible plants *Clitoria* sp and *Brassica* sp were extracted and incorporated into ι-carrageenan film as a colorimetric pH sensor film for monitoring food spoilage and its freshness. The color changes of the developed colorimetric sensor film were measured with chromametry and UV-vis spectroscopy, respectively. Experimental results show that colorimetric pH sensor film demonstrated statistically significant differences (p < 0.05) between CIE-L*a*b* coordinates color system indicated that the developed colorimetric sensor film was able to give a gradual change in color over a wide pH range. The color of the colorimetric sensor film also changes discretely and linearly with factors that contribute to food spoilage using shrimp and durian samples. Moreover, the developed colorimetric pH sensor film has the potential to be used as a safe, non-destructive testing and also a flexibly visual method for direct assessment of food freshness indicator during storage.

## 1. Introduction

Food spoilage have a profound impact on security, environment, quality and food safety. In some cases, many foods item were discarded because of perishable before sold. These losses have a major environmental and direct economic impact especially on retailers, food services and consumers if it is not addressed promptly [1,2]. A reduction in shelf life of foods as a result of microbial contamination and an increase in the risk of food-borne illness are driving forces for innovative means to monitor freshness of the foods while enhancing its safety [3]. Therefore, established technique such as gas-chromatography [4], mass spectroscopy [5] infrared spectroscopy [6] magnetic resonance imaging [7] and microbiological analysis [8] has become the method of choice to evaluate the food quality. However, these techniques are bulky, requires bench top instrumentation, costly and time-consuming [9]. Alternatively, a new concept have been developed through the fabrication of intelligent packaging in the form of a food spoilage indicator to monitor quality status.

Real time chromametry measurement based on CIELab system offered a great potential as a measurement tool to assessing food security and often used in the food industry [10,11]. This system is based on a “standard eye” with filters for three primary colors comprising red, green and blue and also has been suggested by Commission International de l′Eclairage (CIE) due to its reliable system for description of color [12]. However, knowledge of quality-indicating metabolites is needed as a main prerequisite in the development of food spoilage indicator [13] particularly for real time chromametry measurement. Typically, these metabolites are substances responsible for food color change produced by bacteria, mold and yeast during their deterioration such as carbon dioxide, sulfur dioxide, ammonia, amine, organic acids, ethanol, toxins or enzymes [14]. Besides, it also can be used as a food marker for commercial freshness indicators [15]. Therefore, several natural and synthetic compounds reported offers particularly useful and inexpensive metabolites indicator that potentially providing direct information on product quality within food product [16].

Previously, some scientists have identified a few synthetic compounds that capable to response through color change in the presence of target volatile compounds such as bromocresol purple [17], methyl red [18], bromophenol blue [19] and chlorophenol red [20]. However, in food applications, these synthetic compounds are unfitted to be used as sensor for food freshness determination because they are harmful to the environment and are associated with allergic, toxic and other harmful reactions [21]. Hence, the pigments extracted from natural resources are expected to be safe and secure in sensor application as it is low toxicity, renewable, convenience and free from contamination [22].

Anthocyanins typically can be found primarily in flower petals, leaves and fruits offering an alternative to synthetic food colorants which unfortunately is undesirable due to health concerns [23]. These types of food colorants, also known as bio-colorants, can be found in some tropical plants such as Nees (Strobilanthes cusia), Red potatoes (Solanum tuberosum), True indigo (Indigofera tinctoria), Marigold (Tagetes erecta) and Turmeric (Curcuma longa) [24,25]. Among these, anthocyanins from Butterfly pea (*Clitoria* sp) and red cabbage (*Brassica* sp) are the few natural food colorant, used widely around the world ranging from drink dye to food due to its fascinating deep blue and red color [26,27]. Previous studies showed that both plants also act as naturally-derived pH dyes for colorimetric bio-indicator and perceived as lower risk to the consumer. In addition, both plants have been successfully used in the development of colorimetric sensor as they provide significant changes on the color spectra of the films at a certain level of food freshness [28,29].

In order to natural dye indicators to work well, they need entrapment matrices to achieve uniform immobilization to stabilize its function. Previously, variety of entrapment matrices have been extensively used for immobilization of anthocyanin based material such as glass beads [30], hydrogel polymeric [31] membranes [32], sol-gel matrices [33] and even cassava starch [28]. Carrageenan is composed of linear polysaccharide chains, with sulfate half-esters attached to the sugar unit has wider industrial applications, especially as food grade materials is one of the most widely used immobilization matrix [34].

Currently, there is very little investigation on the use of mixed natural dyes for the fabrication of colorimetric sensor for the direct and in-contact food quality assessment. Thus, the idea to use a mixture of anthocyanin extracted from two edible plants and incorporated them into one single platform matrices is expected to have benefit for detecting of food freshness because of its non-toxic nature. Additionally, mixed-dye indicators could be potentially broader the range of color change as compared with a single indicator, thus enabling a wider pH range response. Therefore, in this study, the development and characterization of colorimetric pH sensor film for food spoilage assessment based composite film was performed with a mixed natural dye containing an extract from the edible parts of the *Clitoria* sp and *Brassica* sp plants and ι-carrageenan film. Anthocyanins contain in *Clitoria* sp and *Brassica* sp is expected to exhibit a reversible change in molecular structure as the pH of solutions change from acidic to basic. To identify the color change in response to pH, a statistical evaluation was performed to correlate the color response of the sensor film (L*, a*, b* and C*) with the measured pH values of the samples.

## 2. Material and Methods

### 2.1. Materials and Reagents

The *Clitoria* sp flowers were plucked from the MARDI, Serdang planting area. The *Brassica* sp was bought from the local market at Kajang, Malaysia. ι-carrageenan was purchased from J&K (China; degree of deacetylation, 18–20%; molecular weight, 100,000–300,000). Three types of buffer solutions were used to test the color schemes of the colorimetric sensor film consist of hydrochloric acid-potassium chloride buffer (pH 1–2), citrate buffer (pH 3–8) and carbonate-bicarbonate buffer (pH 9–10). In the case of pH 11–13, the combination of hydrochloric acid-sodium hydroxide was used and their pH values were measured using a digital pH meter (CyberScan pH 510, EUTECH). The pH values of shrimp flesh and durian pulp are determined in triplicate on the homogenate sample with distilled water using a digital pH meter. All solutions were prepared using ultrapure water obtained from a Millipore purification system.

### 2.2. Extraction of Anthocyanins from Clitoria sp and Brassica sp

To extract anthocyanins from *Clitoria* sp, aqueous extraction method was done according to the procedure as described in Kungsuwan et al. [35] with some modification. Firstly, 40 g of the petals were dissolved in 100 mL distilled water about 30 min and grind them in a blender for 30 s.

While the extraction method for *Brassica* sp has been carried out using the method proposed by Devarayan & Kim [36] with some modification. The sample was extracted by taking 50 g of grinding *Brassica* sp and soaked in 100 mL of 80% ethanol. After that, it kept under constant stirring using shaker (150 rpm, 12 h) at room temperature in the dark condition.

Then, both samples were filtered separately through two layers of muslin cloths to remove any coarse particles. Each of the filtrates samples was centrifuged (Heraeus # 7590 centrifuge) at 4293.12 g for 10 min to remove the fine suspended particles. Subsequently, 100 mL of each clear extracts were concentrated under vacuum using Buchi, Rotavapor R-3 at 50 °C to filtrate volume of about 20 mL. Finally, the concentration of anthocyanin in the extracted solution was determined by Liquid Chromatography Mass Spectrometry of Quadrupole Time-Of-Flight (LCMS-QTOF-MS/MS). The respective concentrated extracts samples consist of *Clitoria* sp and *Brassica* sp were stored at −19 °C until ready to use.

### 2.3. Fabrication of Colorimetric pH Sensor Film

The film for colorimetric pH sensor was prepared by slowly dissolved ι-carrageenan in distilled water under continuous stirring with a magnetic stirrer until the powder was completely dissolved. A concentrated dye extract containing anthocyanin from the *Clitoria* sp and *Brassica* sp extract mixture was homogenized in filmogenic suspension with ratio of 1:1. The obtained solutions were then poured into a series of Petri dishes. To control the film thickness, the quantity of each film-forming solution is always fixed. Drying is done by using an oven at a temperature of 37 ± 1 °C for 18–24 h. The films obtained from the plate were removed and stored in desiccators. Response optimization of the three raw materials such as CaCl_2_ concentration, mixed natural dye concentration and the amount of matrix polymer ι-carrageenan were carried out after the fabrication of colorimetric sensor film. The preparation method of control ι-carrageenan film is similar to colorimetric pH sensor film but without added of CaCl_2_ and mixed natural dye.

### 2.4. Film Characterization for Colorimetric pH Sensor

#### 2.4.1. Spectroscopy Analysis

The UV-vis spectra of single extracts, mixed natural dye and colorimetric sensor film in the different pH buffer solutions (pH 1–13) were analyzed using a UV-Vis Spectrophotometer (Varian Carry 100) in the range of 380–800 nm. While Fourier transform infrared (FTIR) spectra were recorded by Perkin-Elmer spectrophotometer (Spectrum 400 Model) with resolutions of 4 cm^−1^ in the wavenumber range of 4000–650 cm^−1^. All the spectra presented were obtained in transmission mode.

#### 2.4.2. Color Measurements

The color value of the sensor was measured with a chromameter (Konica Minolta, Chroma Meter CR 400, Tokyo, Japan). The values of the rectangular coordinates (L*, a*, b*), where (L*) is lightness, a* is the degree of redness or greenness and b* is the degree of yellowness or blueness were recorded to calculate the perceptual correlate chroma value, C* (Equation (1)) [37] and total color differences, ΔE* (Equation (2)) [38] by using equation below:

C*= (a*^2^ + b*^2^) ^½^ L (1)

ΔE* = [(ΔL*)^2^ + (Δa*)^2^ + (Δb*)^2^] ^½^ (2) (2)

where: ΔL* = L* − L0*; Δa* = a* − a0*; Δb* = b* − b0* (L0*, a0* and b0* are the color parameters of the reference standard using a white standard plate provided by Minolta with color coordinates of L*_standard_ = 94.79, a*_standard_ = −0.42 and b*_standard_ = 3.44.

#### 2.4.3. Repeatability and Reproducibility Study

Repeatability and reproducibility analysis was performed by referring Zhang et al. with modification [32]. The sensing film was immersed in a pH 4 buffer solutions then analyzed in the range of 380–800 nm of wavelength. These steps were repeated 5 times using different film and buffers for repeatability but the similar film and buffers were used for reproducibility analysis. Then, the whole procedure was repeated for buffer solution of pH 7 and 12. The percentage of RSD was calculated using the following Equation:

RSD (%) = (Standard deviation/average) × 100(3)

### 2.5. Food Application

#### 2.5.1. Sample Preparation 

The portion of shrimp was put on the polystyrene tray while polypropylene tray has been used for durian sample. To ensure a closed space, both trays were packed into packaging material of oriented nylon polyethylene (Ony/PE) with 0.10 mm of thickness. Then the sensor film was placed at the headspace of each package before sealing properly. The shrimps and durian samples were stored at ambient temperature (28 ± 1 °C; 70–85% RH) during storage.

#### 2.5.2. Total Volatile Base Nitrogen (TVB-N) Analysis

The determination of TVB-N content of shrimp flesh was carried out using the micro-diffusion Conway method [39]. The analysis consists of a trichloroacetic acid extraction followed by alkalinisation and incubation at 37 °C for 60 min. Finally, the total volatile bases in a boric acid solution were titrated with hydrochloric acid and their concentration expressed as N mg/100 g sample.

#### 2.5.3. Microbiological Test 

Total plate count, yeast and mold counts and lactic acid bacteria (LAB) count of the samples (durian and shrimp) was performed according to the ICMSF methods for microbiological examination of foods [40]. A serial 10-fold dilution was prepared and 1 mL of appropriate decimal dilutions was poured on plate of total plate agar (PCA), potato dextrose agar (PDA) and Man Rogosa Sharpe (MRS) agar to determine total plate count, yeast and mold and lactic acid bacteria, respectively. The microbiological analysis was conducted in duplicates and the results were expressed as logarithm of colony forming units (log CFU/g) of sample.

### 2.6. Statistical Analysis

ANOVA was selected to compare the mean differences of the samples using statistical analysis system (SAS, Version 9.4) software. Meanwhile, Duncan′s multiple range test was used to compare the differences for each change in pH solution [41]. The values were considered significantly different when p < 0.05. 

## 3. Results and Discussion

### 3.1. Determination of Anthocyanin Content in Samples of Clitoria sp and Brassica sp.

In this study, the extract of *Clitoria* sp and *Brassica* sp were evaluated using Liquid chromatography–mass spectrometry (LCMS-QTOF-MS/MS). The results found that 12 different compounds of anthocyanin were successfully detected where ternatins were identified as the largest anthocyanin groups from Clitoria ternatea flowers comprising B1, B2, C1, D1 dan D2 types (Table 1). These results are consistent with the previous report showing that a variety of ternatin anthocyanin compounds were accumulated in Clitoria ternatea flowers [42]. Ternatins have become the largest monomeric anthocyanin which comprise delphinidin 3-O-(6″-O-malonyl)-β-glucoside derivatives substituted at both the 3′ – and 5′ – OH groups with glucose or acylated glucose chains of various lengths [43].

Subsequently, the results of chromatographic analysis showed four different compounds of tentative anthocyanins were detected in extracted solution of *Brassica* sp, as shown in Table 1B. Contrary to the *Clitoria* sp, the most dominant anthocyanins detected in Brassica sp are cyanidin with four different moieties. The similar result have shown in previous studies where cyanidin is the most common in *Brassica* crops with quantitative differences among species and crops within the species [44,45].

### 3.2. Colour of Mixed and Single Natural Dyes Containing Anthocyanin in Buffer Solution (pH 1–13)

**Figure 1:**
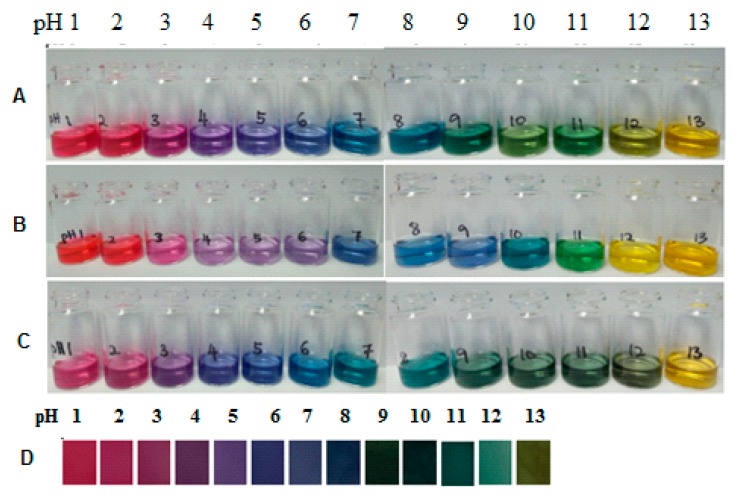
The color of solutions of (**A**) mixed natural dye (*Brassica* sp + *Clitoria* sp); (**B**) *Brassica* sp; (**C**) *Clitoria* sp extract) and (**D**) Colorimetric pH sensor film in different pH buffers solution (pH 1–13).

Figure 1 shows the color changes in different sample consists of mixed natural dye solution (*Brassica* sp + *Clitoria* sp); (b) *Brassica* sp; (c) *Clitoria* sp; and (d) the developed colorimetric pH sensor film in different pH buffers (pH 1–13). Clearly, the mixed natural dye (Figure 1A) was capable of producing distinct color changes over a wide range of pH values compared to single extract (Figure 1B,C). As it can be seen, the mix solution presented a red color for the pH solution below pH 4.0 and changed to purple around pH 5.0. Then, it turned from blue to green in the pH range from 6.0 to 11.0. Finally, yellow at pH solution was above 12.0. Similarly, the developed colorimetric pH sensor film showed that the color variation is dependent on pH changes and the its color changes were easily distinguishable one from another at different pH (Figure 1D).

### 3.3. UV-vis Spectra of the Mixture and Single Extracts of Natural Dye in Various pH Ranges

**Figure 2:**
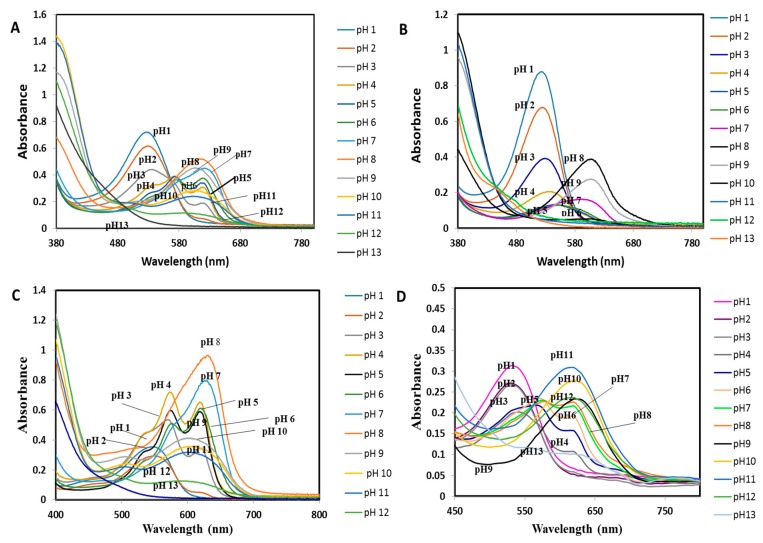
UV-vis spectra of the non-immobilized dye extracts of (**A**) mixed natural dyes (*Brassica* sp *+ Clitoria* sp); (**B**) *Brassica* sp; (**C**) *Clitoria* sp; and (**D**) Colorimetric pH sensor film in different buffers (pH 1–13).

Corresponding to color changes in Figure 1, UV-vis measurement was employed to investigate absorption spectra for each mixture solution in the region from 380 nm to 800 nm. It is pertinent to point out that the observed spectra produced in this study relied on the anthocyanin contents whether the flavylium cation or quinonoidal base form of which is a major component in this system [46]. The UV-vis results indicates that the absorption spectra of mixture pH (Figure 2A) are able to produce maximum absorbance peak at different wavelengths compared a single extract (Figure 2B,C. As can be seen, UV absorption was measured at 527 nm and 530 nm, respectively for pH 1 and 2. Moreover, two maximum absorption peaks and a small shoulder peak were observed at a wavelength of 571 nm and 619 nm (pH 4), 574 nm and 615 nm (pH 5) and 577 nm and 618 nm (pH 6), respectively. These two maximum peaks are representing the quinonoidal base and anionic quinonoidal base. While small shoulder peak could be attributed to the flavylium cation species. This characteristic is commonly exhibited by acylated B-ring substituted anthocyanin as can be seen in a previous study [47]. Furthermore, as the pH increased above 7.0, the maximum absorption of the peak shifted to a value more than 600 nm. This shift also known as bathochromic shift commonly found in anthocyanins [48].

Furthermore, the resulting absorption spectra for the colorimetric pH sensor film in different pH buffers solution (pH 1–13) demonstrated the maximum absorption display wavelength (λ_max_) at 530 nm for pH lower than 4.0 (Figure 2D). These can be concluded that when the absorbance value decreases, pH also tends to increases and resulting in the color of the film changing from red to purple. Moreover, a new peak at 570 nm appeared when the pH of the sensing film increased to 5, 6 and 7. The absorption peak at 620 nm was found at pH 8.0 and above. Meanwhile, the absorption intensity of 620 nm increased when the solution pH increased from pH 8.0 to 10.0 and the color of sensing film changed from blue to green as shown in Figure 1B. However, no absorption peak wavelength is found in the wavelength range of 450–800 nm at pH 13.0. It may be caused by the degradation of anthocyanin compound at extreme pH values. According to Giusti & Wrolstad [25], the isolated anthocyanins are highly unstable and very susceptible to degradation where their stability is very much dependent on pH value.

### 3.4. Chroma Colour Parameter Values

The chromametry value (L*, a*, b*, C* and ΔE*) obtained in this study was further analyzed to see the variation in their mean at different pH buffer (significant level p < 0.05). As can be seen in Table 2, the lightness (L*) of the film was found decreased significantly (p < 0.05) as decreased the pH value. The degradation of color lightness can be attributed to the degradation of antrocyinin at higher pH as suggested in previous studies [49].

Subsequently, a* value exhibited significant difference at different range pH buffer, suggesting that the colorimetric pH sensor film was graded towards red within the buffer range of pH 1 to pH 3. While the red color could be assigned to the flavylium cation form of the anthocyanin structures that appears when anthocyanin in strongly acidic medium [50]. However, the colorimetric pH sensor film showed a negative values for the parameter a* starting at pH 9.0 (−9.41 ± 0.02) and being more distinct at pH 11.0 (−14.72 ± 0.27) where color changes can be seen on the colorimetric pH sensor film from red to bright green color.

Furthermore, the highest negative values for parameter b* obtained indicates the presence of blue color visually displayed at pH 6.0, followed by pH 7.0 and pH 8.0 with values of −20.01 ± 0.02, −18.36 ± 0.29 and −18.25 ± 0.01, respectively. Meanwhile, the chroma (C*) of the film shows decrease with increasing in pH value (p < 0.05) where the sensing film gave the brighter color in acidic pH buffer solution (pH 1–2). However, color intensity gradually fade away whereas the chromatic (C*) value of film are decreases in the alkaline pH range and being more evident at pH 9.0 (11.06 ± 0.01). These phenomena can be related to the deprotonated of cynadin molecules occurring at high pH to form an anion which contributed to the cynadin degradation in the film [50]. The results from the data obtained conclude that 83.08% of the data show significant differences (p < 0.05) for color parameters (L*, a*, b*, C* and ΔE*) in different buffers. This result demonstrated that the developed film owing a significant color variation enabling color variability to the human eye. Particularly, the color parameters a* and C* showed the highest number of significant difference between their values and pH. Thus, both of these color parameters could be used as a primary assessment of the color change of the sensor in response to the pH changes.

In addition, correlation studies have been carried out between each chromametric parameter and different pH range to find out the most significant parameter correlated in the particular pH range. In this studies, the highest correlation coefficients (R^2^) value was obtained in selected pH ranged as shown in Table 3 indicates that colorimetric pH sensor film would have better color distinction in those pH. In general, the results showed that all of the chromametrics parameters (L*, a*, b*, C*, ΔE*) of the colorimetric pH sensor film are well correlated with the pH values (Table 3). Among these, the a* value had displayed a strong correlation with widest pH range (R^2^ = 0.97391), followed by C*(R^2^ = 0.94694), ΔE*(R^2^ = 0.93431 at pH 1–5), b* (R^2^ = 0.86177), ΔE* (R^2^ = 0.93431 at pH 6–10) and L* value (R^2^ = 0.76366) respectively. Therefore, it can be concluded that a* parameter implies the best color parameter can be used as food spoilage assessment. However, since all the color parameters (L*, a*, b*, C* and ΔE*) are quantitatively measured and display a good correlation with pH, others parameter is also possible to be use as food monitoring along with suggested parameter.

### 3.5. Repeatability and Reproducibility Studies

**Figure 3:**
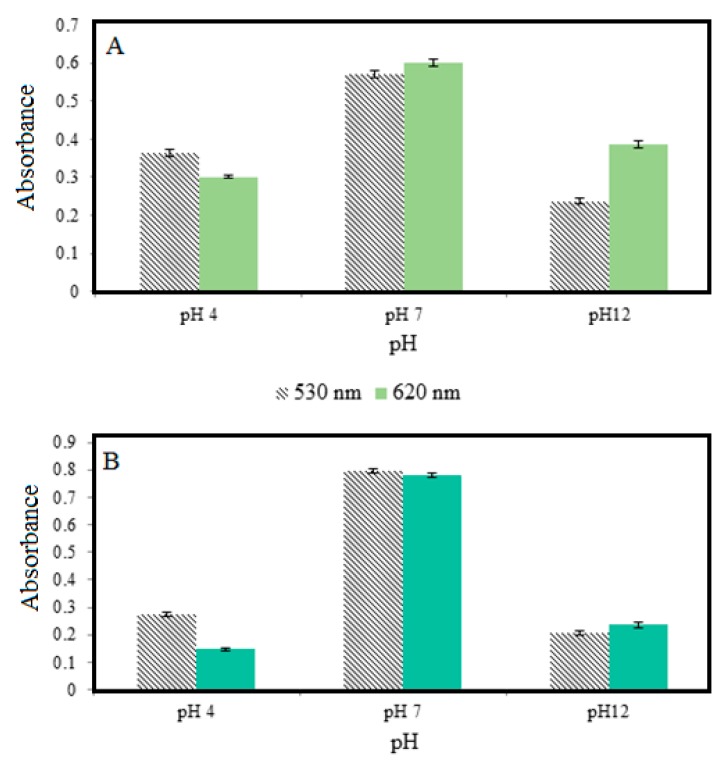
(**A**) Graph of repeatability and (**B**) reproducibility of colorimetric pH sensor film in different buffer solutions.

The repeatability of colorimetric pH sensor film was studied using three different buffers consists of pH 4, pH 7 and pH 12 and measured using UV–vis spectroscopy by referring at UV adsorption values. The results obtained are shown in Figure 3A for both wavelengths 530 nm and 620 nm. As a result, the relative standard deviation (RSD) values were obtained at pH 4, pH 7 and pH 12 was 2.26%, 1.64% and 2.85% (absorption at 530 nm wavelength), respectively. While RSD values obtained at the same pH was 1.40%, 1.55% and 2.06% for the 620 nm wavelengths, respectively. All of these RSD values can be described as a low and acceptable which indicates homogeneously dispersed between a mixture of natural dye and thickness of the colorimetric pH sensor film.

Furthermore, Figure 3B shows the results of reproducibility studies of colorimetric pH sensor film were carried out on the same condition as repeatability studies. As a result, the RSD values for pH 4, pH 7 and pH 12 was determined to be 2.47%, 1.25% and 3.28%, respectively for the absorption at 530 nm wavelengths, while values of 4.62%, 1.08% and 4.05% for the absorption wavelength at 620 nm. This result shows that the resulting RSD value is higher than the repeatability and indirectly indicates that the colorimetric pH sensor film has better repeatability than its reproducibility.

### 3.6. Sensor Response of Food Samples

#### 3.6.1. Sensor Response of Food Samples during Storage

Two types of food sample were use as real samples to demonstrated the effectiveness colorimetric pH sensor film. In this study, fresh shrimp and durian samples were tested using the colorimetric pH sensor film in food spoilage evaluation stored at ambient temperature (28 ± 1 °C; 70–85% RH).

**Figure 4:**
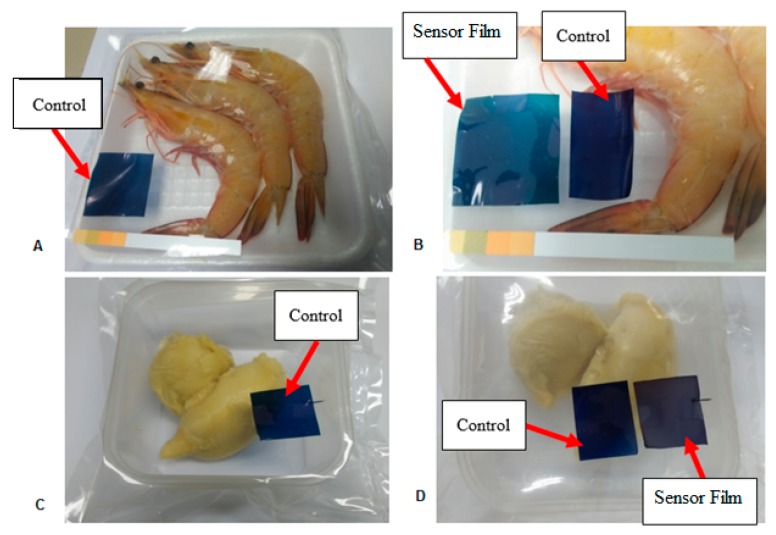
Application of sensing film for monitoring—(**A**) shrimp freshness at 0 h (i) and after 2.5 h; (**B**) stored at ambient temperature; (**C**) durian freshness at: 0 days; (**D**) and after 4 days (ii) stored at ambient temperature.

Table 4 shows the changes in color parameters (L*, a*, b*, C* and ΔE*) of the colorimetric pH sensor film throughout storage at ambient temperature for shrimp and durian samples, respectively. For shrimp samples, the L* value is found decreased with the increasing storage time, resulting the film became darker. Subsequently, the negative a*value is detected on the colorimetric pH sensor film in the pH 9 buffer. These results demonstrated the ability of colorimetric pH sensor film to change its color that occurred at this pH value ascribed the result to the the chemical reaction between anthocyanin compounds in the form of anhydro bases and the spoilage metabolites [51]. While, the value of b* showed decreased throughout storage periods exhibited diminishing of the blue color on the colorimetric pH sensor film (Figure 4A,B).

On the other hand, the results on the durian sample also displayed decreases in L* value pattern throughout 6 days of the storage and the film became darker until the end of the storage time. Subsequently, the same pattern was observed at a* value in a first two days where the color colorimetric pH sensor film turned to distinct red after 6 days of durian storage. However, the pattern changes to increase at day 4 of the storage time. The results of this outcome is difficult to be explain however it could be due to anthocyanins nature properties where in nature they are highly unstable and are susceptible to degradation according to the material of food used. Moreover, negative values of b* (blue) decreased to −2.93 ± 0.04 at day 4 of the storage time where the appearance of dark purple are visible on the colorimetric pH sensor film (Figure 4C,D). This change may be due to the degradation of food metabolites leading to a pH decrease in headspace. Similar results are shown on the color intensity value (C*) showed decreases in pattern with increases the storage time for both samples. This pattern could be due to the change of a* and b* values since both parameters have a great influence on the changes of color intensity (C*). While, total color difference (ΔE*) also increased as increases the storage time with the value obtained was 56.27 ± 0.84 on the first day and keep increasing until 70.67 ± 0.27 on day 4.

#### 3.6.2. Correlation between pH Samples with Colorimetric Parameters for Shrimp and Durian Samples

**Figure 5:**
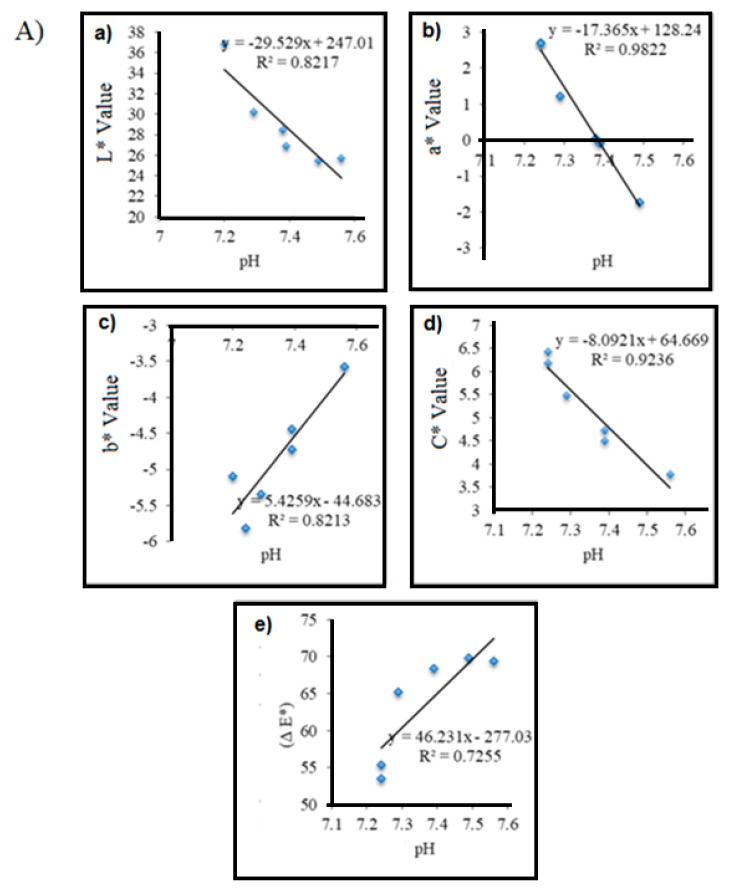

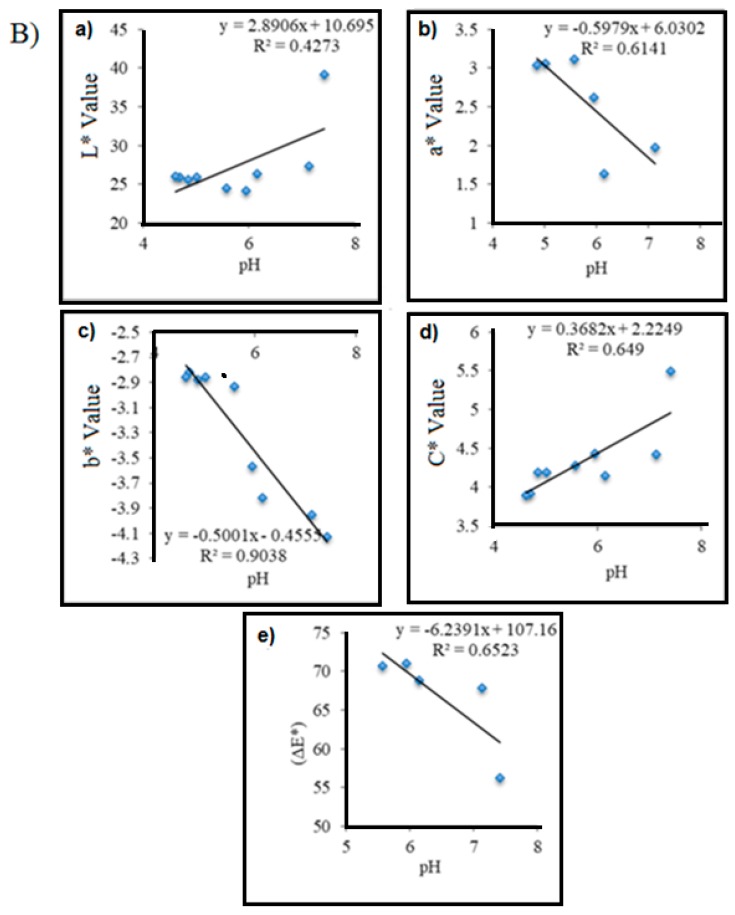
Sample of (**A**) shrimp samples (**B**) durian sample based on correlation studies between color parameter of colorimetric pH sensor film and pH values.

Figure 5A shows the correlation graph between color parameter of the developed colorimetric pH sensor film (L*, a*, b*, C*, ΔE *) and pH value for fresh shrimp samples stored at 28.1 ± 1 °C for 4 h. In this study, the change of a* value displays a strong relationship with the pH value compared to others color parameters (Figure 5B). These can be seen when the pH of the shrimp sample increased, negative value of b * also increase which also indicates the color of the film turned to greenish blue after 2.5 h of storage. Hence, it can be concluded that, the negative value for a * on the colorimetric pH sensor will increase as the freshness of the shrimp sample decrease.

While, Figure 5B represent correlation graph between color parameter (L*, a*, b*, C*, ΔE*) and pH value for durian sample was stored at ambient temperature for 8 days. As a result, the value of b* showed a highest correlation than other studied color parameters where the increases acidity of the durian sample (low pH value) exhibited the value of b * (referring to blue) decreased. Therefore, it can be concluded that the parameter color of b* value on the developed colorimetric pH sensor will decrease as there is decreases of quality durian sample freshness. The results also showed that the colorimetric pH sensor changed to purple dark on the 4th day of storage with a pH value detected was about 5.5 ± 0.02 which is considered not accepted as fresh edible durian [52].

#### 3.6.3. Correlation Study of the Colorimetric pH Sensor Film on the Freshness of Package Shrimps and Durians Stored at Ambient Temperature (28 ± 1 °C)

**Figure 6:**
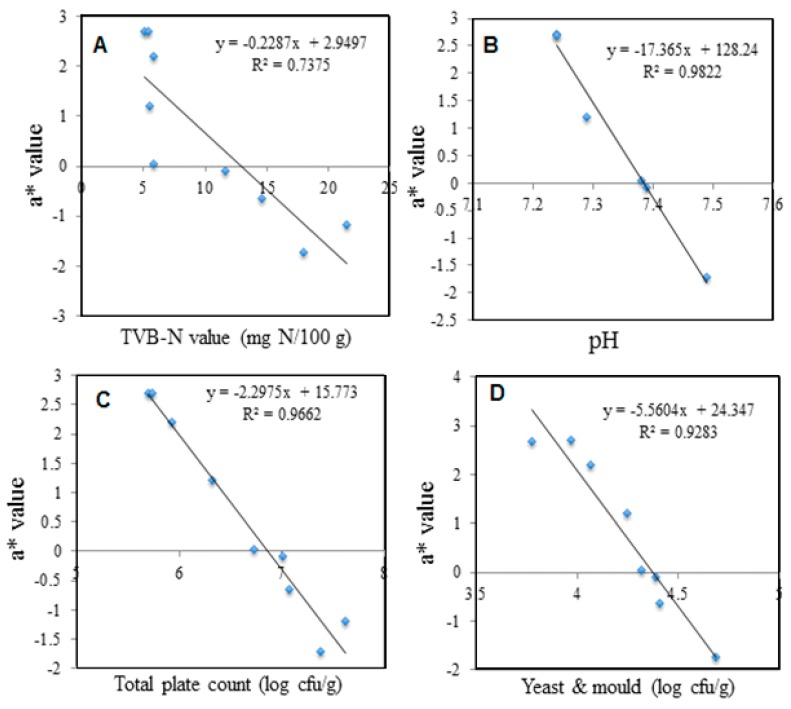
Correlation between a* value of colorimetric pH sensor film with parameter analysis of shrimp samples—(**A**) TVB-N concentration; (**B**) pH value—(**C**) Total plate count (TPC); (**D**) Yeast and mold counts.

Correlation studies have been conducted between chromametrics parameter (L*a*b*c) and food spoilage parameter (TVB-N, pH, TPC, Yeast & mold and LAB) to determine whether the correlation between these two variables is significant or not. In general, the value of chromametrics parameter (L*a*b*c) was found to have a good correlation with the food spoilage parameter (Table 5). Among these, a* value showed correlate well to all food spoilage parameters involved compared to another chromametrics parameter throughout 4 h storage time for shrimp samples. Specifically, an increases pattern was observed for −a* (negative value refers to green) as increased of TVB-N contents (Figure 6A), pH (Figure 6B), total plate counts (TPC) (Figure 6C) and yeast & mold (Figure 6D), respectively, which represents the deterioration of food quality with the increasing storage time.

**Figure 7:**
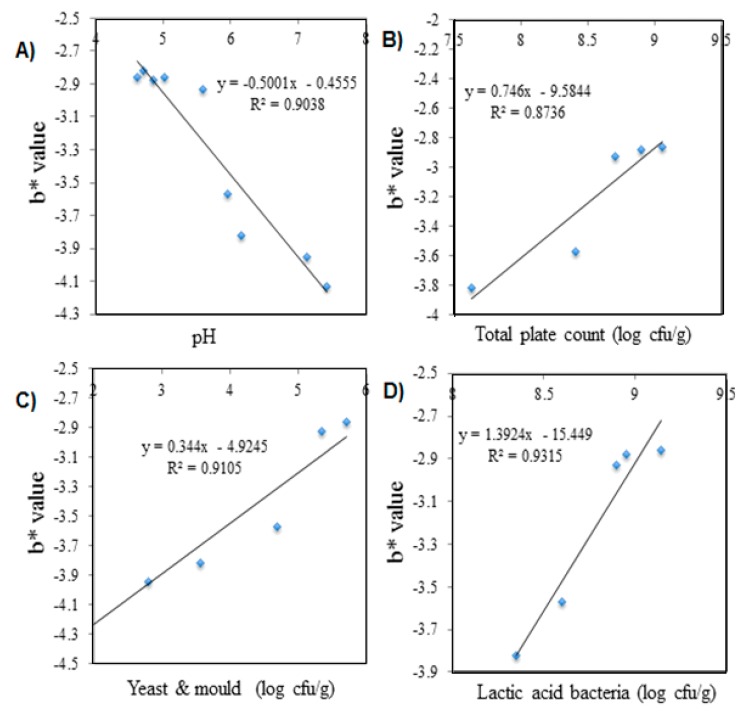
**Correlation** between b* value of colorimetric pH sensor film and parameter analysis of durian samples—(**A**) pH value; (**B**) Total plate count—(**C**) Yeast & mold counts; (**D**) Lactic acid bacteria counts (LAB).

On the other hand, durian samples also showed fairly strong relationship between values of L*, a*, b*, C* and food spoilage parameters (Table 5). Among these, b* value (refers to blue) significantly correlated with all the food spoilage parameter consists of pH (Figure 7A), total plate counts (TPC) (Figure 7B), Yeast and mold (Figure 7C) and lactic acid bacteria (LAB) (Figure 7D) by displaying strong correlation coefficients (R^2^ > 0.90) respectively. As it can be seen, the depreciation -b* value indicates less blue color was observed in colorimetric pH sensor film can be indicated the deterioration of the durian quality within 6 days of storage time.

Furthermore, the change in negative values of a* and b* explain the existence of correlation between the metabolites that caused food spoilage produced during storage time due to increase of TVB-N content, pH values and microbiology contents. Based on this result, two color parameters (a* and b*) have shown their suitability for quantitative measurement of packaged shrimp and durian samples quality using colorimetric pH sensor film developed by chromametry methods.

## 4. Conclusions

In this study, colorimetric pH sensor film containing anthocyanin was developed by incorporating mixed natural dyes extracted from *Brassica* sp and *Clitoria* sp, using ι-carrageenan as immobilization platform. This colorimetric sensor was developed for the purpose of monitoring food spoilage shows the distinct color changing in ranged of pH between 1.0 to 10 in studied buffer solution. The ability of the developed colorimetric pH sensor film to shows color changes on shrimp and durian sample provides a simple way to express the quality of food could offer an efficient alternative approach for monitoring spoilage degree of food samples with potential for the development of intelligent packaging giving direct information on food quality. In future, the proposed colorimetric pH sensor film could be used along with colorimetric readout that function as a sensor that quickly responds and enabling customers to make decisions easily.

## Figures and Tables

**Table 1:** Extract compound detected using LCMS-QTOF-MS/MS for (**A**) *Clitoria* sp; (**B**) *Brassica* sp.

	Compound Detected	Retention Time (Min)	[M]+ (m/z)	Formula
**(A)**	***Clitoria*** **sp**			
1	Ternatin B1	29.45	1945.5063	C_90_ H_97_ O_48_
2	Ternatin B2	29.37	1637.4229	C_75_ H_81_ O_41_
3	Ternatin C1	19.43	1329.3364	C_60_ H_65_ O_34_
4	Ternatin D1	35.67	1783.4616	C_84_ H_87_ O_43_
5	Ternatin D2	32.41	1475.3745	C_69_ H_71_ O_36_
6	Delphinidin-3-glucoside	21.21	627.1696	C_27_ H_31_ O_17_
7	Delfinidin	26.08	303.0492	C_15_ H_11_ O_7_
8	Peonidin-3-O-glucoside	31.01	625.1806	C_28_ H_33_ O_16_
9	Peonidin 3-sambubioside	20.19	595.1663	C_27_ H_31_ O_15_
10	3-O-beta-d-glucoside 5-O-(6-coumaroyl-beta-d-glucoside	34.92	757.1982	C_36_ H_37_ O_18_
11	Cyanidin 3-(6″-caffeyl-2‴-sinapylsambubioside)-5-(6-malonylglucosid)	24.23	1197.2949	C_55_ H_57_ O_30_
			
			
12	Cyanidin 3-O- [ 2-O- (6-O-E-coumaroyl-beta-d-glucopyanosyl) ] -{6-O- [ 4-O- (6-O-E-coumaroyl-beta-d-glucopyranosyl) -E-caffeoyl ] -beta-d-glucopyranosyl}-5-O-beta-d-glucopyranoside	32.04	1197.2949	C_66_ H_69_ O_33_
**(B)**	***Brassica*** **sp**			
1	Cyanidin 3,3′,5-tri-O-glucoside	13.88	773.2151	C_33_ H_41_O_21_
2	Cyanidin 3-(6″-sinapylsophoroside)-5-glucoside	21.13	979.2714	C_44_H_51_O_25_
3	Cyanidin 3-(diferuloylsophoroside) 5-glucoside	34.39	1125.3103	C_53_H_57_O_27_
4	Cyanidin 3-O-[beta-d-glucopyranoside]-7,3′-di-O-[6-O-(sinapyl)-beta-d-glucopyranoside	34.82	1185.3305	C_55_H_61_O_29_

**Table 2:** Color parameters changes (L*, a*, b*, C* and ΔE*) of colorimetric pH sensor film at different buffers.

pH	L*	a*	b*	C*	ΔE*
1.0	62.23 ± 0.03 ^B^	47.23 ± 0.06 ^A^	5.24 ± 0.04 ^B^	47.51 ± 0.06 ^A^	57.74 ± 0.06 ^A^
2.0	64.30 ± 0.04 ^A^	43.79 ± 0.04 ^B^	0.50 ± 0.04 ^C^	43.79 ± 0.04 ^B^	53.78 ± 0.05 ^C^
3.0	61.89 ± 0.01 ^C^	35.87 ± 0.04 ^C^	−4.41 ± 0.03 ^E^	36.14 ± 0.04 ^C^	49.60 ± 0.04 ^E^
4.0	60.75 ± 0.05 ^D^	24.74 ± 0.05 ^D^	−12.32 ± 0.02 ^I^	27.64 ± 0.05 ^D^	45.16 ± 0.02 ^G^
5.0	59.24 ± 0.20 ^E^	19.09 ± 0.14 ^E^	−17.12 ± 0.17 ^J^	25.64 ± 0.21 ^E^	45.47 ± 0.23 ^G^
6.0	57.91 ± 0.24 ^G^	11.02 ± 0.05 ^F^	−20.01 ± 0.02 ^L^	22.84 ± 0.04 ^F^	45.18 ± 0.22 ^G^
7.0	59.44 ± 0.48 ^E^	7.49 ± 0.26 ^G^	−18.36 ± 0.29 ^K^	19.83 ± 0.37 ^G^	42.28 ± 0.67 ^I^
8.0	54.08 ± 0.03 ^I^	6.92 ± 0.02 ^H^	−18.25 ± 0.01 ^K^	19.52 ± 0.02 ^H^	46.71 ± 0.04 ^F^
9.0	43.20 ± 0.03 ^J^	−9.41 ± 0.02 ^K^	−5.82 ± 0.01 ^F^	11.06 ± 0.01 ^M^	53.18 ± 0.03 ^D^
10.0	40.86 ± 0.03 ^K^	−8.66 ± 0.02 ^J^	−9.85 ± 0.02 ^H^	13.12 ± 0.01 ^L^	56.15 ± 0.03 ^B^
11.0	56.07 ± 0.56 ^H^	−14.72 ± 0.27 ^M^	−7.98 ± 0.18 ^G^	16.75 ± 0.31 ^I^	42.83 ± 0.71 ^H^

Average values (n = 5) with the same superscript within a column are not significantly different at 5% level (p > 0.05). Number of data demonstrated Significant difference: L* = 9; a* = 13; b* = 11; C* = 13; ΔE* = 8. Total significant = 54. Significant data percentage = 83.08%.

**Table 3:** Quantitative data on the linear relationship between color parameters (L*, a*, b*, C* and ΔE*) and pH.

Correlation Parameter (y)	pH Range (x)	Linear Correlation Relationship (n = 5 Data Points)	R^2^
L*	1–10	y = −2.3293x + 69.201	0.76366
a*	1–10	y = −6.5453x + 53.807	0.97391
b*	1–8	y = −3.6944x + 6.0336	0.86177
C*	1–9	y = −4.2672x + 49.555	0.94694
∆E*	1–5	y = −3.316x + 60.298	0.93431
∆E*	6–10	y = 3.284x + 22.428	0.81002

**Table 4:** Parameters changes (L*, a*, b*, C* and ΔE*) of sensing film for packaged shrimp and durian stored at ambient temperature (28 ± 1 °C).

Storage Time (h/day)	L*	a*	b*	C*	ΔE*
**Shrimp samples**					
1.0 h	36.81 ± 0.62	2.20 ± 0.22	−5.09 ± 0.17	5.55 ± 0.20	58.66 ± 0.62
2.0 h	28.47 ± 0.69	0.04 ± 0.08	−5.13 ± 0.26	5.13 ± 0.26	66.87 ± 0.76
2.5 h	26.91 ± 0.65	−0.09 ± 0.22	−4.72 ± 0.38	4.72 ± 0.38	68.37 ± 0.63
3.0 h	26.31 ± 0.73	−0.64 ± 0.10	−4.44 ± 0.41	4.49 ± 0.40	68.93 ± 0.70
4.0 h	25.73 ± 0.20	−1.18 ± 0.10	−3.57 ± 0.79	3.77 ± 0.74	69.43 ± 0.25
**Durian samples**					
0 day	39.18 ± 0.77	3.62 ± 0.22	−4.13 ± 0.31	5.49 ± 0.40	56.27 ± 0.84
2 day	26.38 ± 0.61	−1.63 ± 0.10	−3.82 ± 0.20	4.15 ± 0.23	68.82 ± 0.62
4 day	24.50 ± 0.28	3.12 ± 0.08	−2.93 ± 0.04	4.28 ± 0.08	70.67 ± 0.27
6 day	25.58 ± 0.11	3.04 ± 0.04	−2.88 ± 0.14	4.19 ± 0.13	69.59 ± 0.12

**Table 5:** Correlation studies between color changes (L*, a*, b*, C* and ΔE*) of sensing film with spoilage parameters for shrimp and durian samples stored at ambient temperature (28 ± 1 °C).

Spoilage Parameters	L*	a*	b*	C*	ΔE*
**Shrimp samples** **Correlation (n = 5) with**					
**TVBN**	R2 = 0.5300	R2 = 0.7375	R2 = 0.7025	R2 = 0.6127	R2 = 0.5271
	(y = −0.7604x +39.259)	(y = −0.2287x +2.9497)	(y = 0.0887x −5.886)	(y = 0.1025x + 6.2913)	(y = 0.7393x + 56.35)
**pH**	R2 = 0.8217	R2 = 0.9822	R2 = 0.8213	R2 = 0.9236	R2 = 0.7255
	(y = −29.529x + 247.01)	(y = −17.365x + 128.24)	(y = 5.4259x −44.683)	(y = −8.0921x + 64.669)	(y = 46.231x – 277.03)
**TPC**	R2 = 0.8872	R2 = 0.9662	R2 = 0.6887	R2 = 0.7752	R2 = 0.8856
	(y = −8.636x + 88.487)	(y = −2.2975x + 15.773)	(y = 0.7707x – 10.063)	(y = −1.0117x + 11.92)	(y = 8.4114x + 8.3878)
**Yeast & mold**	R2 = 0.6648	R2 = 0.9283	R2 = 0.8842	R2 = 0.8693	R2 = 0.8544
	(y = −11.452x + 80.281)	(y = −5.5604x + 24.347)	(y = 1.7352x – 12.432)	(y = −2.0813x + 14.14)	(y = 21.044x – 25.782)
**Durian samples**					
**Correlation (n = 5) with**					
**pH**	R2 = 0.4273	R2 = 0.6141	R2 = 0.9038	R2 = 0.649	R2 = 0.6523
	(y = 2.8906x + 10.695)	(y = −0.5979x + 6.0302)	(y = −0.5001x − 0.4555)	(y = 0.3682x + 2.2249)	(y = −6.2391x + 107.16)
**TPC**	R2 = 0.6485	R2 = 0.642	R2 = 0.8736	R2 = 0.6582	R2 = 0.6446
	(y = −1.5199x + 38.567)	(y = 0.7261x − 3.609)	(y = 0.746x − 9.5844)	(y = −0.1583x + 5.5111)	(y = 1.4857x + 56.899)
**Yeast & mold**	R2 = 0.5995	R2 = 0.8216	R2 = 0.9105	R2 = 0.5706	R2 = 0.5955
	(y = 2.7226x + 39.181)	(y = 0.4882x + 0.3074)	(y = 0.344x −4.9245)	(y = −0.2745x + 5.5356)	(y = 2.6603x + 56.303)
**LAB**	R2 = 0.6473	R2 = 0.828	R2 = 0.9315	R2 = 0.6606	R2 = 0.6438
	(y = −1.2407x + 36.329)	(y = 1.827x – 13.367)	(y = 1.3924x – 15.449)	(y = −0.1226x + 5.2682)	(y = 1.2132x + 59.084)
